# Relationship between Longitudinal Continuity of Primary Care and Likelihood of Death: Analysis of National Insurance Data

**DOI:** 10.1371/journal.pone.0071669

**Published:** 2013-08-22

**Authors:** Henri Leleu, Etienne Minvielle

**Affiliations:** 1 Health Economics and Outcome Research, Public Health Expertise, Loos, France; 2 Research Unit–Management of Healthcare Organization, Ecole des Hautes Etudes en Santé Publique – Gustave Roussy, Villejuif, France; CUNY, United States of America

## Abstract

**Background:**

Continuity of care (COC) is a widely accepted core principle of primary care and has been associated with patient satisfaction, healthcare utilization and mortality in many, albeit small, studies.

**Objective:**

To assess the relationship between longitudinal continuity with a primary care physician (PCP) and likelihood of death in the French general population.

**Design:**

Observational study based on reimbursement claims from the French national health insurance (NHI) database for salaried workers (2007–2010).

**Setting:**

Primary care.

**Patients:**

We extracted data on the number and pattern of visits made to a PCP and excluded all patients who did not visit a PCP at least twice within 6 months. We recorded age, gender, comorbidities, social status, and deaths.

**Main outcome measures:**

The primary endpoint was death by all causes. We measured longitudinal continuity of care (COC) with a PCP twice a year between 2007 and 2010, using the COC index developed by Bice and Boxerman. We introduced the COC index as time-dependent variables in a survival analysis after adjustment for age, gender and stratifying on comorbidities and social status.

**Results:**

A total of 325 742 patients were included in the analysis. The average COC index ranged from 0.74 (SD: 0.35) to 0.76 (0.35) (where 1.0 is perfect continuity). Likelihood of death was lower in patients with higher continuity (hazard ratio for an increase in 0.1 of continuity, adjusted for age, sex, and stratified on comorbidities and social status: 0.96 [0.95–0.96]).

**Conclusion:**

Higher longitudinal continuity was associated with a reduced likelihood of death.

## Introduction

Continuity of care (COC) is a widely accepted core principle of primary care. Its benefits are thought to include a better physician-patient relationship, increased compliance with physicians' instructions, fewer hospital admissions, higher patient satisfaction rates, and reduced costs [1] [2] [3]. However, it is seldom defined explicitly. The most commonly cited model covers information availability from one health encounter to the next (information continuity), consistency of patient care (management continuity), and the ongoing relationship between patient and provider (interpersonal continuity) [4] [5].

Interpersonal continuity is fundamental in the primary care sector where practitioners have a care-giving responsibility and build up a relationship of trust with their patients [6]. It includes longitudinal COC, i.e. visits to as few practitioners as possible over time, and the quality of the patient-practitioner relationship [7] [8]. Measures for evaluating the patient-practitioner relationship are scarce. Most studies are based on patients' experience and are thus limited in size because of the burden of data collection [9] [10] [11] [12]. On the other hand, several measures are available to evaluate longitudinal COC from routinely available data [4] [7] [13]. Consultations with a practitioner are measured either at various time points (visit measures) or throughout medical care (individual measures) [14]. Measurable attributes of longitudinal COC are concentration (proportion of visits with a given practitioner), dispersion (number of practitioners consulted), distribution (distribution of visits to practitioners), or sequence (whether same practitioner from one visit to the next) [8].

Epidemiological studies and small clinical trials have associated longitudinal COC with patient satisfaction, healthcare utilization, and mortality, mostly in elderly patients and in patients with chronic conditions [15] [16] [17] [18] [19]. To our knowledge, however, there are no studies on the association between longitudinal COC in primary care and mortality in a large sample of the general population. Using the French national health insurance (NHI) reimbursement database, we examined the association of longitudinal COC in the primary care sector and likelihood of death between 2007 and 2010.

## Methods

### Ethics Statement

In France, confidentiality approval only is needed from the *Commission nationale de l'informatique et des libertés* (CNIL) for non-interventional observational studies; ethical approval is not mandatory (Law n° 2004–800 on bioethics, Aug. 6, 2004). CNIL approval for our data source had been obtained previously (CNIL AT/CPZ/SVT/JB/DP/CR05222O (Jun. 14, 2005); DP/CR071761 (Aug. 28, 2007)). Informed consent was not required as data were anonymized.

### Setting

The primary care sector in France chiefly involves primary care physicians (PCPs) with few consultant specialists or other health professionals. Private practice is the mainstay of primary care. Patients can choose to visit any PCP for a consultation and pay an upfront fee-for-service which is partially reimbursed by French NHI. Since 2006, to improve COC, patients have to designate a “usual” PCP but can change this usual PCP as often as they please. Should they visit a PCP other than their usual PCP, the NHI reimbursement rate is reduced except if it is an emergency visit to a PCP. Payment is automatically waived for certain listed chronic conditions (e.g. HIV, cardiovascular diseases, cancer, psychiatric diseases, and diabetes) after the PCP has put in a request to the French NHI, but any chronic condition is eligible for the fee waiver as long as it is justified.

### Study population

We used a sample from the NHI reimbursement database for employees (CNAM-TS) corresponding to about 0.8% of the French population [20]. The database contains all reimbursements made for care covered by French NHI and can be used to identify visits to any PCP. Patients who were affiliated to the CNAM-TS from January 2007 through to July 2010, and who were alive in June 2007, were eligible for the study. As the study focussed on longitudinal COC, we excluded all patients with no regular contact with a PCP, i.e. patients with a greater than 6-month interval between visits to a PCP.

### Outcome measures

Patients were followed from June 2007 to July 2010. The primary endpoint was death by all causes (the database documents month of death). Patients alive after July 2010 were considered censored for the primary endpoint.

We assessed longitudinal COC using the COC index developed by Bice and Boxerman which measures how often a patient saw the same physician over a given time period [21]. The COC index can assume any value between 1 (the patient always saw the same physician) and 0 (the patient never saw the same physician). It is given by formula (1) where n_j_ is, in the present case, the number of visits to PCP i and n is the total number of visits during the time period.
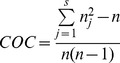
(1)


We chose the COC index as a measure of longitudinal COC for two main reasons: (i) it is independent of the total number of visits, thus reducing the risk of bias caused by an association between repeated visits and disease burden [22] [23]; (ii) it is considered to be an appropriate system level measure of longitudinal COC [8] [24].

The COC index was assessed over 6 months on the basis of visits to PCPs only. We excluded visits to specialist physicians, hospital consultants, and hospital emergency departments which are much less frequent, provide a different service than primary care and thus, cannot be considered as breaks in primary care longitudinal COC. The 6-month time frame was chosen because it is the longest possible length of time for which a drug can be prescribed in France without visiting a physician for a prescription renewal. The COC index for each patient was assessed over the 6 preceding months, at inclusion (June 2007) and twice yearly (Jan. 2008, Jul. 2008, Jan. 2009, Jul. 2009 and Jan. 2010). If a patient visited a PCP less than twice over 6 months, the COC index was considered missing.

Covariates were age at inclusion, gender, comorbidities at inclusion and every 6 months (obtained from hospital data and from payment waivers for chronic conditions), social status, and number of visits to a PCP. We used the ICD-10 Charlson Comorbidity index [25] to measure comorbidities. Any reimbursement made during the study by the French NHI coverage scheme for low income patients was used as a proxy for low social status.

### Statistical analysis

The characteristics of included and excluded patients were compared by student t-test for continuous variables and chi-square test for rates. The mean COC index was calculated for each number of visits (from 2 to 10+). The Pearson product-moment correlation coefficient between the COC index and number of visits was also calculated. The association between the COC index and the likelihood of death was tested using Cox' proportional hazards survival regression analysis.

The selection of the variables for inclusion in the final model for survival was based on a step-down procedure. (i) The base model included the covariates age, gender, comorbidities and social status. Age was converted into a 4-level categorical variable: 0–18, 19–40, 41–65 and over 65 years of age. Comorbidities were converted into a 3-level variable (0, 1, or ≥2) and expressed as a time-dependent variable as comorbidity changes over time can impact on likelihood of death. The COC index was introduced as a continuous time-dependent variable using the counting process method, which is based on creating multiple records per patient, each record corresponding to an interval of time during which all covariates remain constant [26] [27]. Variables with a p-value below 0.05 were kept in the base model. Missing COC index values were replaced by the previously available measurement. When none was available, the period was excluded from the analysis. (ii) Interaction variables obtained by testing variable pairs in the base model were kept in the final model when their p-values were below 0.05. (iii) Proportional hazard assumptions were tested for all variables included in the base model using Pearson's correlation between scaled Schoenfeld residuals for the variable and rank survival time. Significant variables (p = 0.05) were excluded from the final model and used to stratify the model.

The association between included variables and likelihood of death was given as a point estimate with a two-sided 95% confidence interval (CI) for the hazard ratio (HR). The HR was calculated for a 0.1 change in the COC index.

### Sensitivity analyses

We performed several sensitivity analyses: (i) The final model was tested by age category and by maximum number of visits over a 6-month period (fewer than 8; 8 or more). (ii) We used lag times of 6 months, 1 year and 2 years, i.e. earlier instead of current COC index values, to assess whether an association with likelihood of death might not be due to a lower end-of-life COC index [26]. We also tested non-replacement of a missing COC index value by the previous value. (iii) We used another index of continuity of care, the usual provider continuity (UPC) index which measures the frequency at which a patient visits his or her usual provider within a given period. Usual provider was defined as the most visited PCP during this period. Like the COC index, the UPC index was introduced into the model as a time-dependent variable. HRs are given for a 0.1 change in UPC index.

We used SAS 9.2 software (SAS Institute). A two-sided p value of 0.05 was considered significant.

## Results

### Study population

Overall, 396 673 patients were eligible for the study of whom 70 931 (17.9%) were excluded from the analysis as not visiting a PCP at least twice over 6 months. Excluded patients were significantly different from included patients: they were younger (31.4% versus 23.6% in the 19–40 age group, p = ), tended to be male (59.1% versus 45.4%, p = ), were more likely to have no comorbidities (95.8% versus 86.2%, p = ) and were less likely to die (3.4% versus 4.8%, p = ). The characteristics of the 325 742 included patients (no missing data) are given in Table 1.

**Table 1:** Population characteristics.

	Eligible	Included	Excluded
	(N = 396 673)	(N = 325 742)	(N = 70 931)
**Age category N (%)**			
0–18 yrs	81 164 (20.5)	66 510 (20.4)	14 654 (20.7)
19–40 yrs	99 095 (25.0)	76 852 (23.6)	22 243 (31.4)
41–65 yrs	142 882 (36.0)	117 599 (36.1)	25 283 (35.6)
65+ yrs	73 532 (18.5)	64 781 (19.9)	8 751 (12.3)
**Gender N (%)**			
Male	189 935 (47.9)	148 007 (45.4)	41 928 (59.1)
Female	206 738 (52.1)	177 735 (54.6)	29 003 (40.9)
**Low social status N (%)**	38 828 (10.5)*	35 799 (11.0)	3029 (7.2) *
**Comorbidities (Charlson index) N (%)**			
0	348 833 (88.0)	280 864 (86.2)	67 969 (95.8)
1	23 492 (5.9)	22 160 (6.8)	1 332 (1.9)
2+	24 348 (6.1)	22 718 (7.0)	1 630 (2.3)
**Deceased N (%)**	18 066 (4.5)	15 643 (4.8)	2 423 (3.4)
**Mean COC index (SD)** †			
July 2007 (N = 197 721 (60.7%))	0.76 (0.35)	0.76 (0.35)	NA
January 2008 (N = 201 378 (61.8%))	0.74 (0.36)	0.74 (0.36)	NA
July 2008 (N = 199 109 (61.1%))	0.76 (0.35)	0.76 (0.35)	NA
January 2009 (N = 204 086 (62.7%))	0.75 (0.36)	0.75 (0.36)	NA
July 2009 (N = 199 107 (61.1%))	0.77 (0.35)	0.77 (0.35)	NA
January 2010 (N = 191 854 (58.9%))	0.74 (0.36)	0.74 (0.36)	NA

COC: continuity of care; SD: standard deviation; NA: Not applicable.

* 28 684 missing values for social status.

† N: number of available COC index values.

### Continuity of care

The percentage of the population with COC index measurements ranged from 58.9% to 62.7% according to 6-month period, with a mean COC index ranging from 0.74 (SD 0.36) to 0.76 (0.35) (Table 1). The mean COC index according to number of visits per 6-month period ranged from 0.74 to 0.77 (Table 2). Median COC index was 1.0 for 2–7 visits and 0.80 for 10 visits or more (Table 2). Pearson's correlation coefficient between the COC index and number of visits was −0.01 (p<0.0001).

**Table 2:** COC index according to number of visits to a PCP.

Visits/6 months		COC index	
	Median	Mean	95%CI
**2**	1.00	0.77	0.77–0.77
**3**	1.00	0.76	0.76–0.76
**4**	1.00	0.74	0.74–0.74
**5**	1.00	0.74	0.74–0.74
**6**	1.00	0.77	0.77–0.77
**7**	1.00	0.77	0.77–0.77
**8**	0.75	0.76	0.76–0.75
**9**	0.78	0.75	0.75–0.74
**10+**	0.80	0.74	0.74–0.73

COC: continuity of care; PCP: primary care physician; CI:confidence interval.

### Likelihood of death

All variables (COC index, age, gender, social status and comorbidities) were highly significantly (p<0.0001) associated with the likelihood of death and were selected for the base model (Table 3). All interactions between base model variable pairs were significant (p<0.05) and were included in the model. The HRs for the variables of the base model, after taking account of interactions, are given in Table 3. In the tests for proportional hazard assumptions, the residuals of the social status and comorbidities variables showed a significant association with the rank survival time (p = 0.004 and <0.0001, respectively). The final model was thus stratified by social status and comorbidities. It included COC index, age, gender and the following interaction variables COC index*age, COC index*gender, and age*sex.

**Table 3:** Hazard ratios recorded for variables included in the survival regression analysis in the base model, after taking account of variable interactions, and in the final model stratified by social status and comorbidities.

		Base model			Account taken of variable interactions			Final model	
	HR	95% CI	p	HR	95% CI	p	HR	95% CI	p
**COC index**	0.94	0.94–0.94	<.0001	0.96	0.96–0.96	<.0001	0.96	0.95–0.96	<.0001
**Age category**			<.0001 *						
0–18	1	0.99–1.01	0.83	1.4	1.38–1.43	<.0001	1.39	1.37–1.42	<.0001
19–40	Ref			Ref			Ref		
41–65	1.07	1.07–1.08	<.0001	1.2	1.19–1.21	<.0001	1.2	1.19–1.21	<.0001
65+	1.16	1.15–1.17	<.0001	1.48	1.45–1.50	<.0001	1.47	1.45–1.49	<.0001
**Gender**			<.0001 *						
Male	Ref			Ref			Ref		
Female	0.88	0.88–0.89	<.0001	0.75	0.74–0.76	<.0001	0.75	0.74–0.75	<.0001
**High social status**	0.86	0.85–0.86	<.0001	0.9	0.88–0.92	<.0001	–	–	–
**Comorbidies (Charlson index)**			<.0001 *						
0	Ref								
1	1.18	1.17–1.19	<.0001	1.37	1.35–1.38	<.0001	–	–	–
2+	1.16	1.15–1.17	<.0001	1.56	1.52–1.59	<.0001	–	–	–

HR: Hazard ratio; CI: confidence interval; COC: continuity of care; Ref: reference.

* Overall p value.

In the final model, a 0.1 increase in COC index was significantly associated with a decreased likelihood of death (HR: 0.96, 95% confidence interval (95%CI): 0.95–0.96, p<0.0001). Belonging to the 0–18, 41–65 and 65+ age categories and being a female were significantly associated with likelihood of death (Table 3).

The results of the sensitivity analyses are given in Table 4. There was a significant association between the COC index and likelihood of death regardless of age category. The association was stronger for the 0–18, 41–65, and over 65 age categories than for the 19–40 age category. The association was also stronger for patients making 7 or more visits within a 6-month period than for those making fewer than 7 visits. When introducing a lag period (i.e. using earlier rather than current COC indices in the model), the COC index remained significantly associated with likelihood of death. Excluding measurement periods with missing COC values rather than replacing missing values by the previous value did not affect the significant association. Using a 0.1 increase in the UPC index instead of the COC index confirmed a significant association between continuity of care and likelihood of death.

**Table 4:** Sensitivity analyses.

		COC index	
	HR	95% CI	p
**Age category**			
0–18 yrs	0.96	0.96–0.97	<.0001
19–40 yrs	0.98	0.97–0.98	<.0001
41–65 yrs	0.94	0.94–0.94	<.0001
65+ yrs	0.91	0.90–0.91	<.0001
**Maximum number of visits**			
<7	0.95	0.94–0.95	<.0001
7+	0.90	0.90–0.90	<.0001
**Time lag**			
6 months	0.96	0.96–0.96	<.0001
1 year	0.96	0.96–0.97	<.0001
2 years	0.98	0.97–0.98	<.0001
**No replacement of missing COC index values**	0.96	0.95–0.96	<.0001
**UPC index**	0.92	0.91–0.92	<.0001

COC: continuity of care; HR: Hazard ratio; CI: confidence intervals; UPC: usual provider continuity.

## Discussion

In our study of a sample of 325 742 patients visiting a PCP at least twice in 6 months and tracked over 3 years, longitudinal COC was associated with a reduced likelihood of death after adjustment for age, gender, comorbidities and social status.

Certain of our present observations, as well as earlier published observations, favour causality between COC and likelihood of death although this cannot be definitively inferred [28]. First, the association was strong and consistent in all the sensitivity analyses we undertook. Second, the association is plausible as visits to the same PCP can lead to a better understanding of patients' health needs and better management. Thus, as expected, we observed a higher protective effect of COC on mortality in age groups whose medical needs tend to be greater and for whom longitudinal COC may matter most, i.e. the oldest (over 65 s) [17] and the youngest (0–18 years). Third, COC has been repeatedly associated in the literature with better quality of care, fewer hospital admissions, and lower mortality [3] [5] [15] [17] [26] [29]. Two randomized trials have shown better outcomes, greater satisfaction, and fewer hospital admissions with improved longitudinal COC although neither trial looked at mortality [18] [19].

Because longitudinal COC was a time-dependent variable in our study, reverse causation between variable and outcome cannot be totally ruled out [30]. However, even if health status might influence COC [26], reverse causation was unlikely to be significant as the association with likelihood of death was reproducible with lagged COC index values. In addition, our study design using different time lags and survival analysis supports the sequence of COC then death.

Although the association between COC index and likelihood of death was stronger for patients making 7 or more visits within a 6-month period than for those making fewer than 7 visits, the correlation between the COC index and the number of visits to a PCP was weak. Patients making 10 or more visits within a 6-month period had a low mean COC index. This rings true. Many visits may signal poorer health and a higher likelihood of death, but the more visits are made, the more difficult it is to consult the same PCP.

The association between continuity of care and likelihood of death was confirmed by using one of several other measures available for measuring COC. The usual provider continuity (UPC) index measures the rate of visits with the usual provider (in the present case, the PCP visited most often). This analysis yielded results similar to those for the COC index, suggesting that the two indices perform similarly [14] [24]. However, unlike the UPC index, the COC index is mathematically independent of the overall number of visits and is thus not subject to utilization bias [7] [8] [24].

Several factors and limitations need to be taken into consideration in the interpretation of our results. First, we excluded 17.9% of eligible patients as they did not visit a PCP at least twice within a 6-month period during the 3 years of the study. These were the younger and healthier individuals within the general population. We preferred excluding these patients than allocating them a 0 or 1 COC index value as this would have introduced a strong bias in the relationship between health status (and thus mortality) and longitudinal COC. As longitudinal COC is of little relevance in healthy patients with no regular visits to a PCP, the exclusions probably had little impact on the interpretation of our study results.

Second, despite the relatively high rate of missing COC index values, their management by replacing them in the final model by previously available values does not seem to have introduced a bias as a sensitivity analysis excluding these substitute values yielded results that were similar.

Third, the number of covariates available in the French NHI reimbursement database was limited. Important potential confounding factors such as age and presence of comorbidities were available but an unmeasured confounding factor may have accounted in part for the observed association between COC and likelihood of death. In addition, we were unable to distinguish between scheduled visits and unscheduled – possibly emergency – visits and were thus unable to determine whether a decline in COC index in patients approaching death was associated with more frequent unscheduled visits.

Our results have important practical implications. They suggest that longitudinal COC should be encouraged, especially in health care systems where patients are free to see the PCP of their choice. In France, in order to enhance longitudinal COC, patients are invited to designate a usual PCP and are financially encouraged to do so but, as our study shows, COC remains low. The reasons why patients see another PCP need to be explored, with a focus on elderly patients and those with complex conditions.

More research is needed in order to further our understanding of how longitudinal COC affects mortality rates. A study of the difficulties experienced by PCPs in caring for unknown patients with chronic conditions might indicate how long-term COC improves PCP knowledge of patients and whether this knowledge can be transmitted in the patient's medical record. Relationships between longitudinal COC and other concepts such as coordination of care also need to be explored.
